# *Vaccinium meridionale* Swartz Supercritical CO_2_ Extraction: Effect of Process Conditions and Scaling Up

**DOI:** 10.3390/ma9070519

**Published:** 2016-06-25

**Authors:** Alexis López-Padilla, Alejandro Ruiz-Rodriguez, Claudia Estela Restrepo Flórez, Diana Marsela Rivero Barrios, Guillermo Reglero, Tiziana Fornari

**Affiliations:** 1 Institute of Food Science Research CIAL (CSIC-UAM)—CEI UAM + CSIC, Madrid 28049, Spain; alexis.lopez@estudiante.uam.es (A.L.-P.); alejandro.ruiz@uam.es (A.R.-R.); guillermo.reglero@uam.es (G.R.); 2 INTAL Foundation Cra 50 G N° 12 Sur 91, Itagüí 050023, Colombia; crestrepo@fundacionintal.org (C.E.R.F.); vidautil@fundacionintal.org (D.M.R.B.)

**Keywords:** supercritical fluid extraction, *Vaccinium meridionale* Swartz, extracts, scale-up

## Abstract

*Vaccinium meridionale* Swartz (Mortiño or Colombian blueberry) is one of the *Vaccinium* species abundantly found across the Colombian mountains, which are characterized by high contents of polyphenolic compounds (anthocyanins and flavonoids). The supercritical fluid extraction (SFE) of *Vaccinium* species has mainly focused on the study of *V. myrtillus* L. (blueberry). In this work, the SFE of Mortiño fruit from Colombia was studied in a small-scale extraction cell (273 cm^3^) and different extraction pressures (20 and 30 MPa) and temperatures (313 and 343 K) were investigated. Then, process scaling-up to a larger extraction cell (1350 cm^3^) was analyzed using well-known semi-empirical engineering approaches. The Broken and Intact Cell (BIC) model was adjusted to represent the kinetic behavior of the low-scale extraction and to simulate the large-scale conditions. Extraction yields obtained were in the range 0.1%–3.2%. Most of the Mortiño solutes are readily accessible and, thus, 92% of the extractable material was recovered in around 30 min. The constant CO_2_ residence time criterion produced excellent results regarding the small-scale kinetic curve according to the BIC model, and this conclusion was experimentally validated in large-scale kinetic experiments.

## 1. Introduction

The genus *Vaccinium* comprises a group of plants that includes up to 450 species with promising biological activities [1]. In different places its berries are being consumed as a part of a rich dietary source of various phytonutrients, including phenolic compounds such as flavonoids, phenolic acids, lignans and polymeric tannins [2]. In a recent study, Abreu et al. have reported medicinal uses for food uses of 36 *Vaccinium* species, mainly from North America, Asia and Europe [3]. The most commonly reported uses of *Vaccinium* extracts are as antioxidants due to their high content of anthocyanins and other antioxidants [4,5], but in addition to the antioxidant activity due to the anthocyanins, there is evidence of their antidiabetic [6], anti-hyperlipidemic [7], anti-tumorigenic [8] and neuroprotective effects [9].

*Vaccinium meridionale* Swartz (Mortiño or Colombian blueberry) is one of the *Vaccinium* species which grows in the Andean region of South America at 2300–3300 m above the sea level. Some authors have reported a cardioprotective effect of products obtained from Mortiño fermentation, suggesting that the consumption of Mortiño or its related products could be of importance not only in the maintenance of health but also in preventing cardiovascular diseases [10,11].

In the last decade, green technologies such as supercritical fluid extraction (SFE) have been used as alternatives to conventional solvent extraction in the recovery of plant extracts containing phenolic compounds, anthocyanins and other antioxidants [12,13]. Carbon dioxide (CO_2_) is widely used for the extraction of natural compounds since it has moderate critical conditions (304.2 K, 7.38 MPa) and it is a colorless, odorless, nontoxic, non-flammable, safe, highly pure and cost-effective solvent [14,15,16]. Due to its low critical temperature, the thermal degradation of natural products and the subsequent generation of undesirable compounds are minimized or avoided. The low temperatures required, the absence of oxygen during extraction, and the advantage of recovering the extract with high purity, free of solvent, contribute to producing plant extracts of superior quality, i.e., better functional activity, in comparison with extracts produced using liquid solvents.

However, in the case of the genus *Vaccinium*, supercritical CO_2_ extractions have been focused on *Vaccinium myrtillus* L. (blueberry), especially on the study of the extraction of phenolic compounds, anthocyanins and proanthocyanidins using ethanol and water as modifiers [17,18]. To our knowledge, no reports are available in the literature regarding the SFE of Mortiño fruits. Hence, the importance of the present study is based on the use of supercritical CO_2_ extraction to recover bioactives from Mortiño, a widely distributed product in Colombia.

In this work, the SFE of Mortiño from Colombia, previously dehydrated until 12% of the moisture content remained and ground until a mean particle size of 240 µm was reached, was investigated in a small-scale extraction cell (273 cm^3^) using different process conditions: pressures of 10 and 30 MPa, temperatures of 313 and 343 K, and CO_2_ flows of 18 and 32 g·min^−1^. Extraction yields obtained were in a range of 1%–3%. Speedy extraction was observed studying the kinetic behavior of the process.

Theoretical and semi-empirical approaches were applied to study the SFE scaling-up to an extraction cell of 1350 cm^3^ capacity. Pressure and temperature were preserved in both small- and large-scale experiments. Also, in order to make a comparison possible, the same particle size was used, as well as bed density and porosity. Two well-known scale-up criteria were tested [19]: maintaining the same linear velocity or maintaining the residence time of the solvent in the SFE bed. The model of Sovová [20] was adjusted to represent the kinetic behavior of the low-scale extraction and then was used to simulate the large-scale conditions and to evaluate the usefulness of the scale-up criteria.

## 2. Materials and Methods

### 2.1. Plant Material

Fresh and mature (between four and six years old plants) berries of *V. meridionale* were manually harvested in the farm “la Guija”, “El Retiro” zone (2300 m altitude above sea level) belonging to the Antioquia region of Colombia. The berries where then transported to the Instituto de Ciencia y Tecnología Alimentaria (INTAL Foundation, Cra. 50 G N° 12 Sur 91, Itagüí, Colombia) where they were washed and disinfected with the organic disinfectant Citrosan^®^ and then ground in a 15 L capacity cutting machine (Cruells, Girona, Spain) at a chopper speed of 1300 rpm. The disintegrated fruits were then arranged in aluminum trays of 40 × 60 cm containing about 1.5 kg of fruit per tray and subjected to a drying process in a forced convection oven (Binder FD115, Tuttlingen, Germany) to 318 K for 48 h. The product was then removed after 24 h and allowed to cool at room temperature of 296 ± 2 K for 4 h. A second size reduction process was performed using the same cutting machine as described before, and the final product with a particle size range of 500 μm–1.0 mm was vacuum packed in foil zipsealed pouches (BOPP/polyamide/LDPE) (Alico A.A., Medellin, Colombia) and sent it to the Universidad Autónoma de Madrid.

The plant material density (*ρ_s_*) was determined using a helium pycnometer Ultrapyc 1200e (Quantachrome, Boynton Beach, FL, USA) and resulted to be of 1441.6 ± 0.4 kg·m^−3^.

### 2.2. Chemicals

Ethanol absolute (99.5% purity) was purchased from Panreac (Barcelona, Spain). The CO_2_ (N-38) was obtained from Carburos Metalicos, S.A. (Madrid, Spain).

### 2.3. Supercritical Fluid Extraction

*V. meridionale* Sw. extracts were obtained using a pilot plant supercritical fluid extractor from Thar Technologies Inc (model SF2000; Pittsburgh, PA, USA) with two extraction vessels of 273 cm^3^ (small-scale experiments) or 1350 cm^3^ (large-scale experiments) of capacity.

The SFE devise comprises a cascade decompression system of two separators with independent temperature (±2 K) and pressure (±0.1 MPa) control. The extraction unit also includes a recirculation system, were the CO_2_ is condensed and pumped up to the desired extraction pressure. The pressure in the extraction cell is controlled by an automated back pressure regulator valve. The CO_2_ flow is measured using a flow meter from Siemens AIS (Model: Sitrans F C Mass 2100 DI 1.5, Nordborgvej, Denmark). The SFE PLC-based instrumentation and controls as well as the rest of the features have been described in detailed in previous works [21,22].

#### 2.3.1. Small-Scale Extractions

First, small-scale kinetic behavior was studied using the 273 cm^3^ cylindrical extraction vessel (internal diameter = 0.043 m; height = 0.188 m) packed with 0.160 kg of ground Mortiño with a mean particle size of 254 μm. The selected extraction conditions were of 30 MPa, 313 K and 32 g·min^−1^ of CO_2_. The first data point was measured after 10 min of extraction, and the rest of the data were collected at intervals of 20 min until completing the total extraction time (180 min).

Then, in order to study the effect of process conditions on the overall extraction yield, small-scale extractions were accomplished using also 0.160 kg of Mortiño but at two different pressures (10 MPa and 30 MPa) and temperatures (313 K and 343 K). The overall extraction time was also set to 180 min and the CO_2_ flow rate was 18 g·min^−1^.

In all experimental assays the supercritical stream was decompressed at a pressure of 6 MPa in both separators and CO_2_ was recirculated during the whole extraction time. The solid, pasty extracts were recuperated and placed in vials. In order to ensure an accurate determination of the extraction yield with time, separators were washed with ethanol which was eliminated afterwards by evaporation at low temperature (313 K) in a rotavapor R210 (Büchi Labortechnik AG, Flawil, Switzerland).

#### 2.3.2. Large-Scale Extractions

Kinetic data were obtained using the 1350 cm^3^ cylindrical extraction vessel (internal diameter of 0.07 m; height of 0.388 m) loaded with 0.800 kg of ground Mortiño. The extraction pressure and temperature were the same as in the small-scale kinetic experiment (30 MPa and 313 K). The CO_2_ flow rate was set to 158 g·min^−1^ according to the results of the BIC model process simulation. The material extracted at 15, 30, 100 and 160 min of extraction was collected from the separators the same way as described above.

### 2.4. Scaling Criteria and Mass Transfer Modeling

The objective of scaling up is to reproduce the kinetic behavior of the extraction curves obtained when using different extraction cells. Bed geometry is very important in SFE and can influence overall yield so as extract composition [23]. In general, extraction vessels are cylindrical and thus length (*L*) and bed diameter (*D*) of the cylinder are the variables which characterize bed geometry. Yet, to make the kinetic comparison possible, certain process variables such as extraction temperature and pressure should be maintained constant in both small- and large-scale extraction vessels. Also, the same particle size, as well as bed density and porosity, should be preserved. Thus, the key point is to determine the solvent flow necessary in the large-scale device (*Q_LS_*) in order to attain similar kinetic profiles.

In this work two engineering approaches were applied to estimate *Q_LS_* as a function of the solvent flow used in the small-scale experiment (*Q_SS_*) and bed geometry. The first criterion was keeping the CO_2_ velocity constant, thus:

(1) $$Q_{LS}={\left(\frac{D_{LS}}{D_{SS}}\right)}^{2}Q_{SS}$$

Equation (1) was obtained considering that the same temperature and pressure (same CO_2_ density) were preserved in both small- and large-scale experiments, and the cross-flow area of the cylindrical extraction vessels is $A=\pi D^{2}/4$.

The second criterion adopted was keeping the solvent residence time (*t*_R_) constant. The *t*_R_ was calculated as the ratio between the bed volume accessible to CO_2_ flow $\left(\pi D^{2}L\varepsilon \rho /4\right)$ and the CO_2_ flow rate (*Q*) [19]:

(2) $$t_{R}=\frac{\pi D^{2}L\varepsilon \rho }{4Q}$$

where *D* is the internal diameter of the extraction vessel; *ε* is the bed porosity and *ρ* is CO_2_ density. Thus, the constant residence time criterion requires that:

(3) $$Q_{LS}={\left(\frac{D_{LS}}{D_{SS}}\right)}^{2}\left(\frac{L_{LS}}{L_{SS}}\right)Q_{SS}$$

In this work, large-scale kinetic experiments were carried out preserving extraction temperature and pressure as those used in the small-scale kinetic assays (313 K and 30 MPa) and using in each experiment the CO_2_ flow rate predicted by Equations (1) or (2).

Several scaling-up criteria were reported and analyzed in the literature, as described by Zabot et al. [24]. In general, there is no single criterion that can be effectively applied to all systems. For example, keeping the same residence time of the solvent inside the SFE bed (Equation (2)) was successfully applied for the SFE of clove buds but did not result adequate for the SFE of vetiver roots [19].

Vegetal matrices are complex and the type of extractable solutes and their location in the raw material affects the kinetics of extraction. For example, volatile oil is rather easy to extract by SFE, and its major part is obtained in the early stage of the extraction. In these cases, the broken and intact cells (BIC) mass transfer model has demonstrated good capability to represent the kinetic behavior [20]. In the BIC model, the solid phase is considered to be comprised by broken and intact cells and thus the total extractable oil is divided by easily accessible solute, which is available on the surface of the broken cells, and poorly accessible solute which is confined in the intact part of the cells.

The following assumptions are considered in the BIC model: temperature and pressure are constant during the whole extraction time; particle size and oil distributions are uniform in the packed bed; void fraction is constant during the extraction; CO_2_ is solute free at the bed entrance; axial dispersion can be neglected (plug flow is assumed). Then, three different extraction periods can be distinguished:

1. The constant extraction rate (CER) period, in which the external surface of the particles is covered with easily accessible solute, and thus the extraction rate is constant during this period and determined by the convective solvent film resistance.
2. The falling extraction rate (FER) period, in which the intra-particle diffusion starts to become important. The remained accessible solute continues to be extracted but also the solute in the intact cells starts to be extracted. Thus, the extraction rate drops rapidly and at the end of this period, all the readily accessible solute has been removed from the vegetal matrix.
3. The diffusion controlled (DC) period, in which only the less accessible solute in intact cells is slowly extracted. Mass transfer is mainly dominated by slow diffusion inside the solid vegetal particles.

The BIC model equations to calculate the mass extracted (*m*) as a function of extraction time (*t*) in the different periods are the following [20]:

CER period:

(4) m=Q Y*[1−exp(−Z)] t

FER period:

(5) $$m=Q\;Y*\left[t-t_{CER}\exp(Z_{w}-Z)\right]$$

DC period:

(6) $$m=m_{SI}\left\{X_{o}-\frac{Y*}{W}\ln\left[1+\left[\exp\left(\frac{WX_{o}}{Y*}\right)-1\right]\exp\left[\frac{WQ(t_{CER}-t)}{m_{SI}}\right]\left(\frac{X_{k}}{X_{o}}\right)\right]\right\}$$

where,

(7) $$Z=\frac{m_{SI}k_{YA}\rho }{Q(1-\varepsilon )\rho_{s}}$$

(8) $$W=\frac{m_{SI}k_{XA}}{Q(1-\varepsilon )}$$

(9) $$Z_{W}=\frac{ZY*}{WX_{o}}\ln\left\{\frac{X_{o}\exp\left[WQ(t-t_{CER})/m_{SI}\right]-X_{k}}{X_{o}-X_{k}}\right\}$$

(10) $$t_{CER}=\frac{m_{SI}(X_{o}-X_{k})}{Y*ZQ}$$

(11) $$m_{SI}=X_{o}F$$

Process parameters required to apply the BIC model are the bed porosity (*ε*), mass (*F*) and density (*ρ_s_*) of feed raw material, CO_2_ density (*ρ*) and mass flow rate (*Q*). Additionally, the solubility of the extract in the supercritical solvent (Y*) and the global extraction yield (*X_o_*) have to be determined to apply the BIC model.

Parameters which are optimized according to the experimental kinetics are the intra-particle solute ratio (*X_k_*) and the fluid phase and solid phase mass transfer coefficients (*k_YA_* and *k_XA_*). The ready accessible solute (*X_p_*) is calculated as the difference (*X_o_* − *X_k_*).

## 3. Results

### 3.1. Small-Scale Extractions

The experimental conditions and yields obtained in the small-scale assays are given in Table 1. The extraction yield was calculated as the ratio between the mass extracted (*m*) and the mass of Mortiño used (*F*). As expected, the extraction yield increase with increasing pressure. With respect to temperature, the experimental results obtained follow the cross-over behavior observed for the solubility of solutes in supercritical CO_2_: at low pressure (10 MPa) the yield decreased with the rise of temperature, but at higher pressure (30 MPa) the yield increased with increasing temperature.

Extraction yields of *Vaccinium meridionale* Swartz (Mortiño) were lower than 3.2% in the range of conditions explored. The supercritical CO_2_ extraction of *Vaccinium myrtillus* (Blueberry) residues was recently reported by Paes et al. [17]. Although species are different, for the sake of comparison, the yields were around 2% from the fresh sample at 313 K and at pressures of 15–25 MPa. Higher yields were obtained (up to 7.6%) from freeze-dried samples but using water and ethanol as CO_2_ cosolvents.

**Figure 1:**
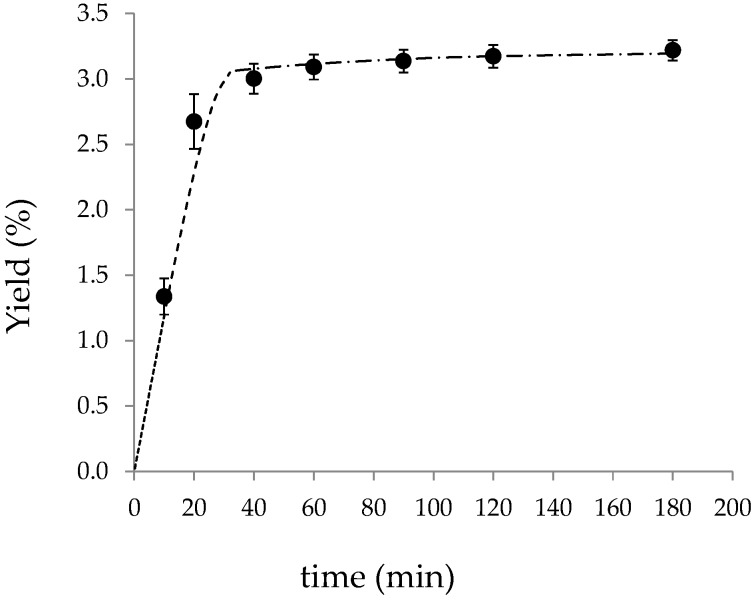
Kinetic behavior of Mortiño SFE at 313 K and 30 MPa in the small-scale extraction cell (273 cm^3^) and with CO_2_ mass flow *Q_SS_* = 32 g·min^−1^. (●) experimental data. Solid lines represent the BIC model fitting: (······) CER period; (- - -) FER period; (- · - ·) DC period.

The kinetics of the overall extraction curve obtained in the small-scale cell at 313 K and 30 MPa is shown in Figure 1. The CO_2_ mass flow was 32 g·min^−1^ during 180 min of extraction, with a solvent-to-raw material ratio of 36 kg·kg^−1^. The experiment was carried out in duplicate; the mean values and standard deviations obtained in the accumulated yield are given in Table 2.

### 3.2. BIC Model Fitting of Small-Scale Experimental Kinetics

The BIC model was used to correlate the kinetic data obtained in the small-scale extraction cell. The Mortiño density (*ρ*_s_) was 1441.6 kg·m^−3^, and the bed porosity was determined on the basis of the corresponding apparent density (592.6 kg·m^−3^) and resulted in *ε* = 0.5890. The CO_2_ density at 30 MPa and 313 K was obtained from thermodynamic tables (*ρ* = 910 kg·m^−3^) [25]. The solubility of the whole extract was estimated as the slope of a theoretical linear behavior of the extraction curve between *t* = 0 and *t* = 10 min (see data in Table 2) and resulted in Y* = 0.006685 kg·kg^−1^ (apparent solubility). The global yield was assessed on the basis of the maximum yield attained (theoretically for *t* → ∞) as 3% above the total amount extracted (*X_o_* = 0.03315 kg·kg^−1^).

Then, the intra-particle solute ratio (*X_k_*) and the mass transfer coefficients in the fluid and solid phases (*k_YA_* and *k_XA_*) were adjusted to reproduce the experimental small-scale kinetic curve. The values obtained are *X_k_* = 0.0018 kg·kg^−1^, *k_YA_* = 0.00490 s^−1^ and *k_XA_* = 0.00016 s^−1^. Table 3 reports the absolute relative deviation (ARD) between the experimental and calculated accumulated yields (the mean ARD was 2.10%).

The optimal intra-particle solute ratio (*X_k_*) was around 10 times lower than *X_p_*, denoting that most of the solute is readily accessible and, thus, during the CER period (*t_CER_* = 5.7 min) more than 23% of the extractable solute was recovered from Mortiño raw material. Moreover, after around 30 min *(t_FER_* = 28.2 min) 94.5% of the extractable material was recovered. Accordingly, the optimal mass transfer coefficient in the fluid phase was 23 times higher than the mass transfer coefficient in the solid phase.

### 3.3. BIC Model Prediction of Large-Scale Mortiño SFE and Comparison with Experimental Large-Scale Extraction

**Figure 2:**
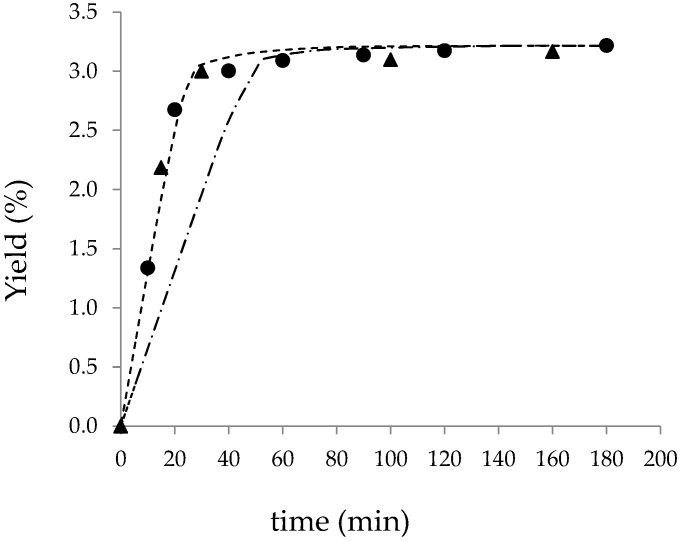
Scaling-up of Mortiño SFE at 40 °C and 30 MPa. Full symbols represent experimental data: (●) small-scale cell with *Q_SS_* = 32 g·min^−1^; (▲) large-scale cell with *Q_LS_* = 158.0 g·min^−1^ (Equation (2)). Lines represent the different BIC model periods: (- ∙ - ∙) *Q_LS_* = 77.6 g·min^−1^ and (- - -) *Q_LS_* = 158.2 g·min^−1^.

The optimized parameters obtained in the BIC model fitting of the small-scale experimental Mortiño kinetics (273 cm^3^ extraction cell, 32 g·min^−1^ CO_2_) were applied to predict the kinetic behavior in the large-scale cell (1350 cm^3^). The CO_2_ density, solid density, apparent solubility and global extraction yield were kept constant. The mass of the raw material (*F_LS_*) was calculated to be 0.800 kg according to the volume of the large-scale cell and to maintain the bed porosity constant (*ε* = 0.5890). The CO_2_ mass flow rate (*Q_LS_*) was calculated using the constant CO_2_ residence time scaling-up criteria (Equation (1)). The *Q_LS_* value that resulted was 158.2 g·min^−1^. Table 4 and Figure 2 show a comparison between the two large-scale kinetics predicted by the BIC model. Certainly, the constant CO_2_ residence time criterion (*t_R_* = 4.58 min) produced the best results of the small-scale kinetic. The predicted *t_CER_* and *t_FER_* were very similar to the values corresponding to the small-scale extraction (see Table 4). Finally, as depicted in Figure 2, the experimental kinetic data obtained from the large-scale extraction also confirmed that maintaining a constant CO_2_ residence time is a valid procedure to scaling-up Mortiño SFE.

Duba and Fiori [26] recently discussed the effect of process parameters on the extraction kinetics of grape seed oil. Regarding the effect of the *D*/*L* ratio, they concluded that while keeping the ratio of the substrate mass to the CO_2_ mass flow rate constant, the lower the *D*/*L* and the lower the specific CO_2_ consumption. In this work, the *D*/*L* was 0.229 and 0.175, respectively, for the small- and large-scale extraction cells. The corresponding substrate mass to CO_2_ mass flow rate (*F*/*Q*) are given in Table 4, together with the specific CO_2_ consumption after 20 min of extraction. The constant *F*/*Q* (constant *t_R_* criteria) denotes almost the same specific CO_2_ consumption to attain the same kinetic behavior, with rather similar *D*/*L* values.

## 4. Conclusions

The supercritical CO_2_ extraction of *Vaccinium meridionale* Swartz was studied to investigate the kinetic behavior, the effect of pressure and temperature on extraction yield and the potential scaling-up criteria.

A high extraction velocity was observed at 30 MPa and 313 K: around 95% of the extractable material was recovered in 30 min of extraction. Moreover, a significant effect of pressure and temperature on the overall extraction yield was determined. In the range of conditions studied, the cross-over behavior of the extraction yield with respect to pressure and temperature was observed.

An accurate representation of the overall extraction curve was obtained with the BIC model. Furthermore, a satisfactory scaling from the small-scale kinetic curve to a five-times-larger extraction vessel was obtained, maintaining constant CO_2_ residence time as the scaling-up criterion.

The studies reported in this work provide preliminary and worthy fundamentals to develop supercritical CO_2_ natural extracts from Colombian *Vaccinium meridionale* Swartz fruit.

## Figures and Tables

**Table 1:** SFE of *Vaccinium meridionale* Swartz (Mortiño). Extraction cell capacity = 273 cm^3^ (0.160 kg of Mortiño); CO_2_ flow = 18 g·min^−1^; Extraction time = 180 min.

Experiment	T (K)	P (MPa)	Yield (%)
1	313	10	1.03
2	343	10	0.08
3	313	30	2.67 *
4	343	30	3.16

* Standard Deviation $=\;\sqrt{\frac{\sum {\left(x_{1}\;-\;x_{2}\right)}^{2}}{2}}$; *x*_1_ and *x*_2_ are values of duplicate experiments (Experiment 3 was the only experiment that was carried out in duplicate).

**Table 2:** Experimental yield (%) obtained in the kinetic study of SFE of *Vaccinium meridionale* Swartz (Mortiño) at 313 K and 30 MPa in the low-scale extraction cell (273 cm^3^). CO_2_ flow = 32 g·min^−1^.

Time (min)	Yield (%)			Standard Deviation (*SD*) *
	Kinetic 1	Kinetic 2	Mean Value	
10	1.24	1.44	1.34	0.14
20	2.53	2.82	2.67	0.21
40	2.92	3.08	3.00	0.11
60	3.02	3.16	3.09	0.09
90	3.07	3.20	3.14	0.09
120	3.11	3.23	3.17	0.09
180	3.16	3.27	3.22	0.08

* $SD=\sqrt{\frac{\sum {\left(x_{1}\;-\;x_{2}\right)}^{2}}{2}}$ being *x*_1_ and *x*_2_ the corresponding values of duplicate experiments.

**Table 3:** BIC model fitting of Mortiño SFE experimental kinetics at 30 MPa and 313 K in a small-scale extraction cell (273 cm^3^).

*T* (min)	Experimental Yield *Y_exp_* (%)	Calculated Yield *Y_cal_* (%)	ARD *
10	1.34	1.30	2.73
20	2.67	2.48	7.39
40	3.00	3.02	0.57
60	3.09	3.14	1.65
90	3.14	3.17	1.01
120	3.17	3.17	0.02
180	3.22	3.18	1.35

* Absolute Relative Deviation = 100 × $\frac{\left|Yexp\;-\;Ycal\right|}{Yexp}$.

**Table 4:** BIC model prediction of Mortiño SFE kinetics at 30 MPa and 313 K in a large-scale extraction cell (1350 cm^3^).

Parameter	Small Scale (273 cm^3^)	Large Scale (1350 cm^3^)	
		Constant *v*	Constant *t_R_*
*F* (g)	160	800	800
*D* (cm)	4.3	6.7	6.7
*L* (cm)	18.8	38.3	38.3
*Q* (g/min)	32	77.6	158.2
*v* (cm/min)	2.42	2.42	4.93
*D*/*L*	0.229	0.175	0.175
*F*/*Q* (min)	5.00	10.30	5.06
*CO*_2_/*F (g*/*g) t* = *20 min*	4.00	1.94	3.96
*t_R_* (min)	4.58	9.32	4.58
*t_CER_* (min)	5.70	5.63	5.63
*t_FER_* (min)	28.2	52.9	28.4
